# BNT162b2 Booster Vaccination Elicits Cross-Reactive Immunity Against SARS-CoV-2 Variants B.1.1.529 and B.1.617.2 in Convalescents of All Ages

**DOI:** 10.3389/fimmu.2022.920210

**Published:** 2022-06-20

**Authors:** Bernd Jahrsdörfer, Matthias Proffen, Judith Scholz, Janina Hägele, Carolin Ludwig, Christiane Vieweg, Aline Grempels, Dorit Fabricius, Ramin Lotfi, Sixten Körper, Guido Adler, Hubert Schrezenmeier

**Affiliations:** ^1^ Department of Transfusion Medicine, Ulm University, Ulm, Germany; ^2^ Institute for Clinical Transfusion Medicine and Immunogenetics, German Red Cross Blood Transfusion Service Baden-Württemberg – Hessen and University Hospital Ulm, Ulm, Germany; ^3^ Department of Pediatrics and Adolescent Medicine, Ulm University Medical Center, Ulm, Germany; ^4^ Medical Faculty, Ulm University, Ulm, Germany

**Keywords:** COVID-19, mRNA vaccine, BNT162b2, neutralization capacity, B.1.1.529, omicron, B.1.617.2, delta

## Abstract

In this prospective observational cohort study we analyzed cellular and serological immune response parameters against SARS-CoV-2 and current variants of concern (VOC) in 147 COVID-19-convalescent and 39 COVID-19-naïve individuals before and after BNT162b2 booster vaccination. No significant differences regarding immunological response parameters were observed between younger and older individuals. Booster vaccination induced full recovery of both cellular and serological response parameters including IFN-γ secretion and anti-spike antibody titers with strong neutralization capacities against wild type SARS-COV-2 and Delta. Surprisingly, even serological neutralization capacity against Omicron was detectable one month after second vaccination and four months before it had been first observed in South Africa. As a result, more than 90% of convalescent individuals exhibited detectable and 75% strong Omicron neutralization capacity after booster vaccination, compared with 72% and 46% of COVID-19-naïve individuals. Our results support the notion that broad and cross-reactive immune memory against SARS-CoV-2 including currently known VOCs can be established by booster vaccination with spike-based mRNA vaccines like BNT162b2, particularly in COVID-19-convalescent individuals of all ages. Nevertheless, especially in COVID-19-naïve individuals future variants escaping the memory immune response may require vaccine approaches such as *inactivated whole virus vaccines*, which include all antigenic components of the virus.

## Introduction

Among the greatest challenges of the current pandemic with severe acute respiratory syndrome coronavirus 2 (SARS-CoV-2) is its ongoing evolution, resulting in variants of interest (VOI) and variants of concern (VOC) with an enhanced capacity for immune escape. The likelihood for the emergence of such variants exponentially rises with the time a host is infected with the virus and the virus is reproduced without being properly controlled by the immune system. Extreme examples for this scenario are immunocompromised patients, e.g. with inherited or acquired immune defects. For example, a meticulous study by Cele and colleagues pursued the evolution of ancestral SARS-CoV-2 persisting for 6 months in a patient with underlying advanced HIV disease (1). This study found that the ancestral virus evolved various mutations which are also known from several VOCs including Omicron. This viral evolution also included a partial escape from BNT162b2-elicited immunity and neutralization by self-plasma. Eventually the patient showed an extensive escape from neutralization by re-infection with the Delta variant.

Although the example above is extreme, an impaired immune response is also a problem of elderly individuals. Therefore, an increased vulnerability to infections makes them a particularly important target population for vaccinations against infectious diseases in general (2). This is also true for COVID-19, for which it is well established that elderly individuals are more susceptible to prolonged and more severe disease courses (3, 4). Efficient vaccination of this population is thus not only in the personal interest of each elderly individual him- or herself, but also in the public interest, since the risk for the evolution of potentially hazardous viral mutations in each individual may be lower, if infections with SARS-CoV-2 are overcome as rapid as possible. This is particularly true in long-term care facilities (LTCFs) with their increased risk for outbrakes (5, 6) and involvement of younger health care workers, who can easily spread viruses with newly accumulated, potentially hazardous mutations. Several studies confirmed the anticipated effectiveness of COVID-19 vaccines among elderly individuals over 60 years of age (6–11). Currently, a particular focus lies on the Omicron variant (B.1.1.529), which was first identified in November 2021 in South Africa and Botswana, and which hosts a so far unprecedented number of mutations, resulting in extensive immune escape and reduced vaccine effectiveness (12).

A plethora of studies meanwhile provide clear evidence that full vaccination and booster vaccination with mRNA vaccines rapidly confers protection against wild type SARS-CoV-2 and a series of VOIs and VOCs, involving both the humoral (6–11, 13) and the cellular (14–16) arms of the immune system. Moreover, SARS-CoV-2 vaccination induces immunological T cell memory (14, 16) and persistent human germinal center responses (17), enhancing the chance for a rapid and successful immune response in case of future infections with SARS-CoV-2 or related viruses. Meanwhile it is evident that neutralizing antibody levels are highly predictive of immune protection from symptomatic SARS-CoV-2 infection (18), so that immunological data translate well into vaccine efficiency. This is also demonstrated by several large studies on vaccine effectiveness, which found substantially lower rates of confirmed COVID-19 by different variants including Omicron, as well as lower rates of severe illness and mortality in participants who received a BNT162b2 booster at least 5 months after the second dose (19–21).

The current study was designed as a follow-up study to our prospective observational cohort study AnCORIm initiated in March 2021 (6). Primary aim was to extend the immune monitoring of fully vaccinated elderly convalescent individuals up to one month after booster vaccination with BNT162b2. End points included several parameters of the T cell response against SARS-CoV-2 including the memory T cell response as well as serological parameters including anti-spike IgG and IgA titers and neutralization capacities against wild-type SARS-Co-2 and the most current variants including Delta and Omicron (Pango lineage BA.1). Particularly the Omicron variant has been shown to extensively escape BNT162b2-induced neutralization in fully vaccinated *COVID-19-naïve* individuals (12). Therefore, a particular focus of our study was serological immunity against Omicron after booster vaccination with BNT162b2 in *convalescent* individuals.

## Materials and Methods

### Study Cohorts

The AnCORIm (“Antikörperantwort COVID-19-Rekonvaleszenter nach Impfung”) study is a prospective observational cohort study, which was initiated in March 2021. The first part of this study was meanwhile completed and results were recently published (6). The second part of the study was planned as a follow-up study after booster vaccination with BNT162b2, 6 months after 2^nd^ vaccination. It was conducted from September 2021 to January 2022 in five long-term care facilities (LTCFs), in which up to 90% of residents and up to 70% of health care professionals (HCPs) had prior SARS-COV-2 infections between March 2020 and February 2021. This COVID-19-convalescent study cohort consisted of 147 individuals. Nobody of these subjects reported any COVID-19-typical symptoms after their 2^nd^ vaccination, or had any history of diseases or medication affecting systemic immunity. As control cohort we included 39 residents of an additional LTCF, who never had a SARS-CoV-2 infection. As in the study cohort, booster vaccination in the control cohort was performed 6 months after 2^nd^ BNT162b2 vaccination. Within the 6-month period between 2^nd^ and booster vaccination all participants had repeated negative nasopharyngeal swabs. Only two healthcare professionals (HCP) had a positive RT-PCR-test, but were asymptomatic. After oral explanation of risks and benefits of vaccination and demonstration of the study protocol, written informed consent was obtained from all participants or the persons appointed to make medical decisions on their behalf. Then, up to 30 ml of blood was collected from all participants directly prior and 30 days after booster vaccination with the BNT162b2 mRNA vaccine. The use of blood from study subjects before and after vaccination was approved by the Ethical Committee at Ulm University. For participant numbers, age and gender characteristics as well as key immunological response parameters of all study cohorts see **Table 1**.

**Table 1:** Age, gender and key immunological response parameters of study cohorts after 3^rd^ vaccination with BNT162b2.

Neutralization capacity > 70%									
Study cohorts	Number (%)	Females	Males	Median age	Wildtype	B.1.617.2	B.1.1.529	Anti-spike IgG	IFN-γ secretion >
				(range)	SARS-CoV-2	(Delta)	(Omicron)	OD ratio > 64 (%)	4x10^3^ mIU/ml (%)
Total vaccinees	186 (100)	140 (75)	46 (25)	63 (20 - 98)	_	_	_	_	_
COVID-19-naive, age > 62	39 (21)	26 (67)	13 (33)	87 (65 - 95)	95	87	46	38	18
COVID-19-convalescent, all ages	147 (79)	114 (78)	33 (22)	62 (20 – 98)	99	98	75	63	55
COVID-19-convalescent, age ≤ 62	73 (39)	61 (84)	12 (16)	50 (20 - 62)	100	100	78	41	62
COVID-19-convalescent, age > 62	74 (40)	53 (71)	21 (29)	84 (63 - 98)	98	97	73	78	50

186 individuals undergoing booster vaccination against SARS-CoV-2 using the mRNA vaccine BNT162b2 were recruited in long-term care facilities including 56% residents and 44% healthcare professionals. 147 individuals were COVID-19-convalescent. 39 residents, who never had a SARS-CoV-2 infection, served as reference cohort. IFN-γ, interferon gamma; OD, optical density; SARS-CoV-2, severe acute respiratory syndrome-coronavirus 2.

### Blood Collection and Cryopreservation

From each donor, up to 15ml blood were collected in serum collection tubes with clot activator (Vacuette, Greiner Bio-One GmbH, Frickenhausen, Germany) for semiquantitative and neutralization testing, and up to 15ml were collected in Heparin-coated glass tubes (BD Vacutainer, Becton Dickinson, New Jersey, USA) for the IFN-γ Release Assay and FACS-based analyses. 250µl-1000µl serum were aliquoted in 2ml cryopreservation tubes (Greiner Bio-One GmbH, Frickenhausen, Germany) after 15min of centrifugation at 1500g and 20°C. Aliquots were cryopreserved at -20°C until further use, the tubes were transferred into -80°C for long-term storage. Plasma from heparinized whole blood samples used for IFN-γ release assays were stored in cryopreservation tubes at -20°C.

### Enzyme-Linked Immunosorbent Assays

For the detection of IgG and IgA to the SARS-CoV-2 S1 (spike) domain and IgG to the SARS-CoV-2 nucleocapsid (NCP) protein, the recently established EUROIMMUN anti-SARS-CoV-2 ELISA assays (22) were run on the fully automated system EUROIMMUN Analyzer I-2P (EUROIMMUN, Lübeck, Germany). In short, the diluted serum samples as well as a calibrator, a positive and a negative control, were transferred onto strips of a 96-well microtiter plate with a coating of either recombinant SARS-CoV-2 spike or NCP proteins and incubated for 60 minutes at 37°C. Wells were washed three times, followed by the incubation with peroxidase-labelled anti-IgG or anti-IgA for 30 min. After another three washing steps, substrate solution was added to the wells and after incubating for 15 - 30 minutes in the dark and adding the stop reagent, the analyzer determined the O.D. values at 420nm and 650nm. O.D. ratios were calculated based on the sample and calibrator O.D. values. For all three analytes, a ratio of < 0.8 was considered to be non-reactive, O.D.-ratios of ≥ 1.1 were meant to be positive. Samples with OD ratios over the cut-off, depending on the calibrator O.D., were prediluted manually in sample buffer at 1:10 - 1:50 and were run again. The results were then corrected according to the dilution factor. For some analyses, an additional high titer cut-off (OD ratio = 64) was used based on the median anti-spike IgG OD ratio in individuals with an Omicron neutralization capacity above 70%.

### Surrogate SARS-CoV-2 Neutralization Test (GenScript, Leiden, The Netherlands)

This blocking ELISA was used as a tool to detect antibodies neutralizing the docking process of the virus (23). These antibodies are inhibiting the interaction between the viral receptor binding domain (RBD) and the cellular angiotensin-converting enzyme 2 (ACE2). Samples and controls were first pre-incubated with horseradish peroxidase (HRP)-conjugated RBD fragment (HRP-RBD) and then transferred to an ACE2-coated microtiter plate. Any HRP-RBD not neutralized by antibodies was captured on the plate. After four washing steps, TMB substrate solution was added to allow HRP to catalyze a color reaction. The stop reagent turned the color from blue into yellow, which was read by a POLARstar Omega microtiter plate reader (BMG Labtech GmbH, Ortenberg, Germany) at 450nm (O.D. 450). The quantity of SARS-CoV-2 neutralizing antibodies is reciprocally correlated with the absorbance of the sample. Positive and negative controls served as internal assay quality controls. A valid test required an O.D. 450 > 1.0 for the negative control and an O.D. 450 < 0.3 for the positive control. The Inhibition score was calculated as follows: Neutralization (%) = (1 - (OD value_sample_/OD value_negative control_) x 100%), with a cut-off value of 30% for detectable and 70% for strong neutralization capacity (23). To measure neutralizing capacities toward the Delta (B.1.617.2) and the Omicron (B.1.1.529) variants of concern (VOCs), appropriate modifications of HRP-RBD were used in the protocol as described above: SARS-CoV-2 spike RBD E484Q and L452R for Delta and the SARS-CoV-2 spike RBD with the following mutations for the Omicron variant: G339D, S371L, S373P, S375F, K417N, N440K, G446S, S477N, T478K, E484A, Q493R, G496S, Q498R, N501Y, Y505H (GenScript, Leiden, The Netherlands and Nanjing, Jiangsu, China).

### IFN-γ Release Assay

This assay detects the secretion of IFN-γ by lymphocytes after specific *ex vivo* stimulation with a mixture of SARS-CoV-2 spike peptides as a measure for T cell immunity after infection and/or vaccination. Using the EUROIMMUN Quan-T-Cell Assay (EUROIMMUN, Lübeck, Germany), 500µl of heparinized whole blood were used for a BLANK tube, the specific SARS-CoV-2 (wild type, Wuhan-Hu-1) TUBE and the STIM tube, containing a mitogen, which is inducing maximal IFN-γ release as a sample-specific internal positive control. All three tubes were incubated for 20-24 hours at 37°C and then centrifuged at 12000g for 10min. The resulting plasma supernatant containing released IFN-γ was stored at 4°C until measurement in an IFN-γ-specific ELISA, or transferred to -20°C for long-term storage. The ELISA was performed on the EUROIMMUN Analyzer I-2P. BLANK, TUBE and STIM samples were diluted at 1:5 or 1:20, according to the programmed assay protocol, and incubated on 8-well strips of a microtiter plate, coated with anti-human IFN-γ antibodies. Six Calibrators with defined IFN-γ concentrations and two controls were carried out in each test run. After the first incubation and washing steps, a second incubation step with biotinylated anti-IFN-γ followed. In a third step, HRP-conjugated anti-biotin and, after a final washing step, substrate was added, which turned blue in presence of bound anti-biotin-HRP. The addition of a stop solution turned the color into yellow, which was detectable at 450nm-620nm. IFN-γ concentrations were calculated by the analyzer on the basis of the calibrators used. The background IFN-γ concentration measured in the BLANK tube was subtracted from each TUBE and STIM sample. If TUBE concentrations exceeded 1x10^4^ mIE/ml, all three corresponding samples, BLANK, TUBE and STIM, were diluted at 1:2 - 1:10 with dilution buffer and measured again. Criteria for valid assay results were BLANK IFN-γ concentrations **≤** 400 mIE/ml and STIM concentrations **≥** 400 mIE/ml. TUBE IFN-γ concentrations < 100mIE/ml were considered as negative, concentrations between 100 and 200 mIE/ml as borderline, and concentrations > 200mIE/ml as positive. Moreover, for some analyses an additional cut-off (4x10^3^ mIU/ml) for strong IFN-γ response was used based on median IFN-γ concentrations before and after 3^rd^ vaccination.

### Intracellular Staining and Flow Cytometry

Heparinized whole blood from COVID-19-convalescent individuals one month after booster vaccination was obtained and stimulated using the EUROIMMUN Quan-T-Cell SARS-CoV-2 kit. To this purpose, 500µl whole blood was added to a negative and a positive control tube as well as a tube containing a mixture of SARS-CoV-2 spike 1 peptides. Then, 8µl Brefeldin A (Brefeldin A Solution 1,000X, BioLegend) was added to each sample, and tubes were incubated for 18 hours at 37°C and 5% CO_2_. After incubation, samples were directly transferred into FACS tubes, washed with PBS (Dulbeccos) and haemolysed using 3.5 ml ACK lysis buffer. Then, cell pellets were permeabilized, fixated and stained with the following fluorescently-labelled antibodies using standard procedures for intracellular staining: anti-human CD4 Vio^®^ Bright B515, anti-human CD8 VioGreen™, anti-human CD3 APC, anti-human IFN-γ PE, anti-human TNF-α PE-Vio^®^ 770 and Anti-Human CD45RO PerCP-Cy5.5, (all from BD, San Jose, California, USA). FACS analysis was performed on a BD FACSCelesta (BD, San Jose, California, USA), data analysis was carried out using FlowJo Software version 10.6.2 (BD, Ashland, Oregon, USA).

## Results

### Characteristics of Study Cohorts and Post-Vaccination Symptoms

For the present study, a total of 186 individuals including 55% residents and 45% healthcare professionals (HCPs) from six long-term care facilities were recruited and the specific immune response monitored after booster vaccination with the mRNA vaccine BNT162b2. 39 individuals (21%) never had a SARS-CoV-2 infection, whereas 147 individuals (79%) were COVID-19-convalescent. Further details on cohort size, age and gender characteristics as well as key immunological response parameters are provided in the Materials and Methods section and are summarized in **Table 1**. Overall, the third vaccination was well tolerated in all participants with no severe side effects observed within 60 min after vaccination or during the following days. The most frequent side effect was injection-site pain in up to 50% of participants, which mainly lasted for less than 12 hours. Other symptoms like fatigue, fever and headache were reported in 20% of residents and 33% in HCPs.

### Full recovery of SARS-CoV-2-Spike-Specific Antibody Titers and Neutralization Capacities in COVID-19-Convalescent Individuals After Booster Vaccination With BNT162b2

**Figure 1:**
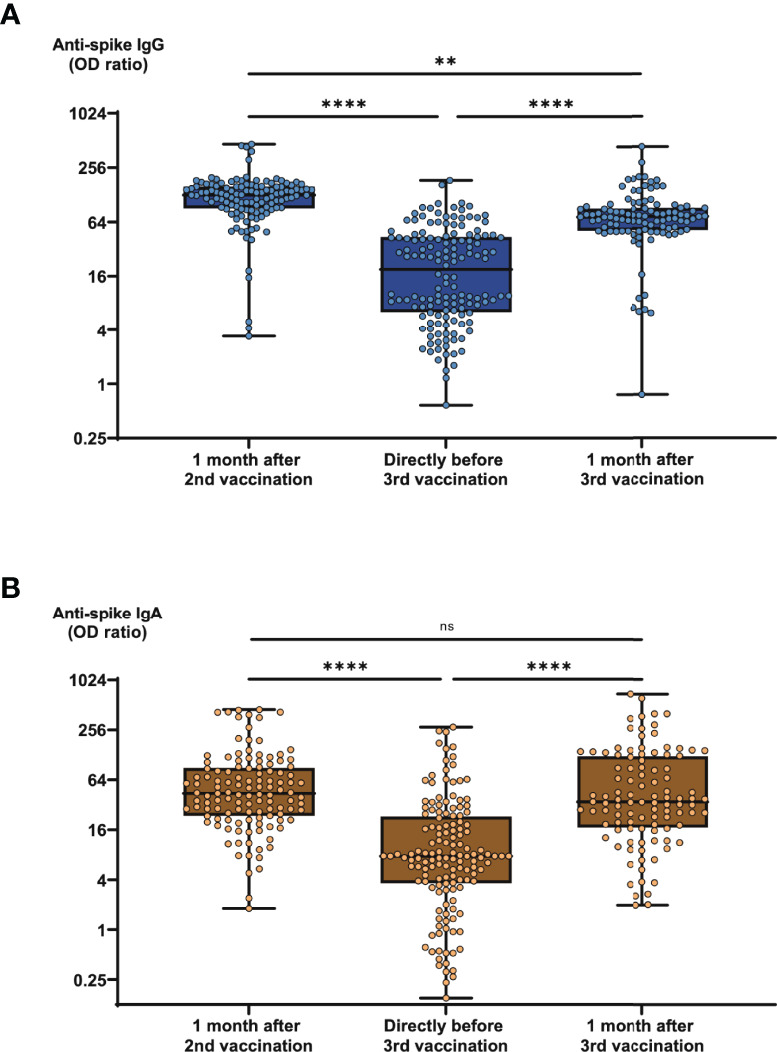
SARS-CoV-2-specific antibody response in COVID-19-convalescent individuals before and after booster vaccination. Serum samples from up to 147 subjects with confirmed COVID-19 history were collected one month after second vaccination with BNT162b2, 6 months after second vaccination (directly before third vaccination), and one month after third vaccination. Samples were then analyzed for anti-spike IgG and IgA titers. Box blots show **(A)** anti-spike IgG titers and **(B)** anti-spike IgA titers at different time points as indicated. Box central horizontal lines indicate medians, box borders represent IQR, whiskers indicate minima and maxima. Significance levels were **p < 0.005 and ****p < 0.00005. IQR, interquartile ranges; OD, optical density; ns, not significant.

In the preceding study, antibody and neutralization titers in COVID-19-convalescent individuals were pursued until one month after second vaccination with BNT162b2 (6). In the present study we extended the observation period by two further time points directly before (corresponding to 6 months after second) and one month after third (booster) vaccination. As shown in **Figure 1**, average SARS-CoV-2-specific antibody titers were subject to a significant waning 6 months as compared to one month after second vaccination. Average anti-spike IgG titers dropped by 77% for IgG and by 70% for IgA within these 5 months (**Figures 1A, B**). On the other hand, booster vaccination induced a strong recovery of antibody titers within 1 month. At this time, average anti-spike antibody titers had significantly increased by 2.8-fold for IgG and by 3.6-fold for IgA, thereby returning to comparable levels as five months before, although anti-spike IgG levels did not fully reach post 2^nd^ vaccination levels (**Figures 1A, B**). As shown in earlier analyses, anti-spike IgG and IgA titers showed a strong and significant correlation among each other (**Supplementary Figure 1A**).

**Figure 2:**
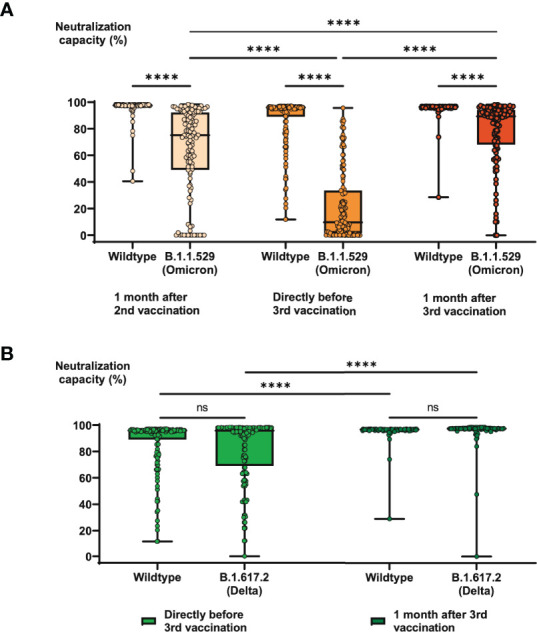
Specific neutralization capacities against current SARS-CoV-2 variants of concern in COVID-19-convalescent individuals before and after booster vaccination. Serum samples from up to 147 subjects with confirmed COVID-19 history were collected one month after second vaccination with BNT162b2, 6 months after second vaccination (directly before third vaccination), and one month after third vaccination. Samples were then analyzed for specific neutralization capacities against wiltype SARS-CoV-2 as well as the variants of concern and B.1.1.529 (Omicron) and B.1.617.2 (Delta). Box blots show **(A)** neutralization capacities against B.1.1.529 compared with wild type and **(B)** neutralization capacities against B.1.617.2 compared with wild type at different time points as indicated. Box central horizontal lines indicate medians, box borders represent IQR, whiskers indicate minima and maxima. Significance level was ****p < 0.00005. IQR, interquartile ranges; ns, not significant.

Of particular interest is the question, whether the serological results described above translate into serological neutralization capacities against SARS-CoV-2, particularly against B.1.1.529 (Omicron) and B.1.617.2 (Delta). Directly before third vaccination, 85% of all COVID-19-convalescent individuals still exhibited strong neutralization capacity (cutoff 70%) against the original wild type virus (**Figure 2A**), compared with 98% of individuals 5 months before (one month after second vaccination). Interestingly, already one month after second vaccination, 56% of individuals already exhibited strong neutralization capacity against Omicron, although at this time point this VOC had not been observed yet. 5 months later, directly before the third vaccination, only 10% of individuals remained with strong Omicron neutralization capacity, which however was significantly boosted by the third vaccination, eventually resulting in 75% of individuals with strong Omicron neutralization capacity one month after booster vaccination (**Figure 2A**). Similar, although not as prominent effects were observed for neutralization capacities against the wild type and the Delta VOC, for which the percentages of individuals with strong neutralization capacity raised from 85% to 99% (wild type) and from 75% to 98% (Delta) after third vaccination (**Figure 2B**).

As in the preceding study, further analyses revealed significant correlations between SARS-CoV-2-spike IgG titers and neutralization capacities (**Supplementary Figure 2**). Surprisingly however, the strongest correlation was found between anti-spike IgG titers and Omicron neutralization capacities, where only 16% of individuals with an IgG OD ratio below the median of 64 exhibited a strong capacity, in contrast to 77% of individuals with an IgG OD ratio above the median of 64 (**Supplementary Figure 2C**). In comparison, correlations were significantly weaker for wild type (88% vs. 98%) and Delta (81% vs. 92%) neutralization capacities (**Supplementary Figure 2A, B**).

### Strong Induction of Anti-Spike Antibody Titers and Neutralization Capacities Against B.1.1.529 and B.1.617.2 After Booster Vaccination With BNT162b2 in All Age Groups

**Figure 3:**
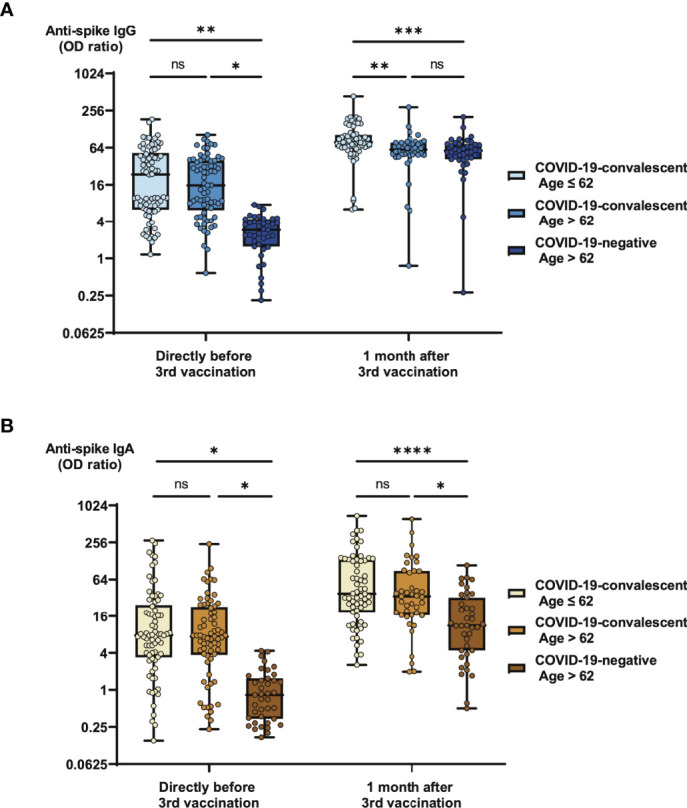
Impact of age and previous SARS-CoV-2 infection on SARS-CoV-2-specific antibody responses before and after booster vaccination. Serum samples from 73 COVID-19-convalescent individuals under the age of 63, 74 COVID-19-convalescent individuals above the age of 62 and 39 individuals above the age of 62, who never had a SARS-CoV-2 infection were collected directly before and one month after booster vaccination with BNT162b2. Samples were then analyzed for anti-spike IgG and IgA titers. Box blots show **(A)** anti-spike IgG titers and **(B)** anti-spike IgA titers before and after vaccination as indicated. Box central horizontal lines indicate medians, box borders represent IQR, whiskers indicate minima and maxima. Significance levels were *p < 0.05, **p < 0.005, ***p < 0.0005 and ****p < 0.00005. IQR, interquartile ranges, ns, not significant; OD, optical density.

As in the first part of our study we were interested whether or not age and previous contact to SARS-CoV-2 have an impact on the efficacy of a booster vaccination with BNT162b2. We therefore evaluated serologic responses directly before and one month after third vaccination in three different cohorts (**Table 1**), consisting of COVID-19-naïve individuals at an age > 62 (median age 87), COVID-19-convalescent individuals at an age > 62 (median age 84) and COVID-19-convalescent individuals at an age ≤ 62 (median age 50). The strongest booster effect was observed in the elderly COVID-19-naïve cohort, where the percentage of individuals with an anti-spike IgG OD ratio > 64 increased from 0% before to 38% after third vaccination (**Figure 3A**). In comparison, the respective percentages in the elderly COVID-19-convalescent cohort raised from 20% before to 78% after third vaccination. Finally, percentages in the cohort of COVID-19-convalescent individuals ≤ 62 years of age ranged significantly lower from 9% to 41% (**Figure 3A**). Development of anti-spike IgA titers followed a very similar pattern as anti-spike IgG titers (**Figure 3B**). As in the first part of our study investigating the effects of the first two vaccinations with BNT162b2 (6), IgA titers were significantly higher in COVID-19-convalescent compared with COVID-19-naïve individuals (16.1-fold before and 3.4-fold after third vaccination). Also in line with our previous study, the age in general appeared to have a rather minor impact on anti-spike antibody titers.

**Figure 4:**
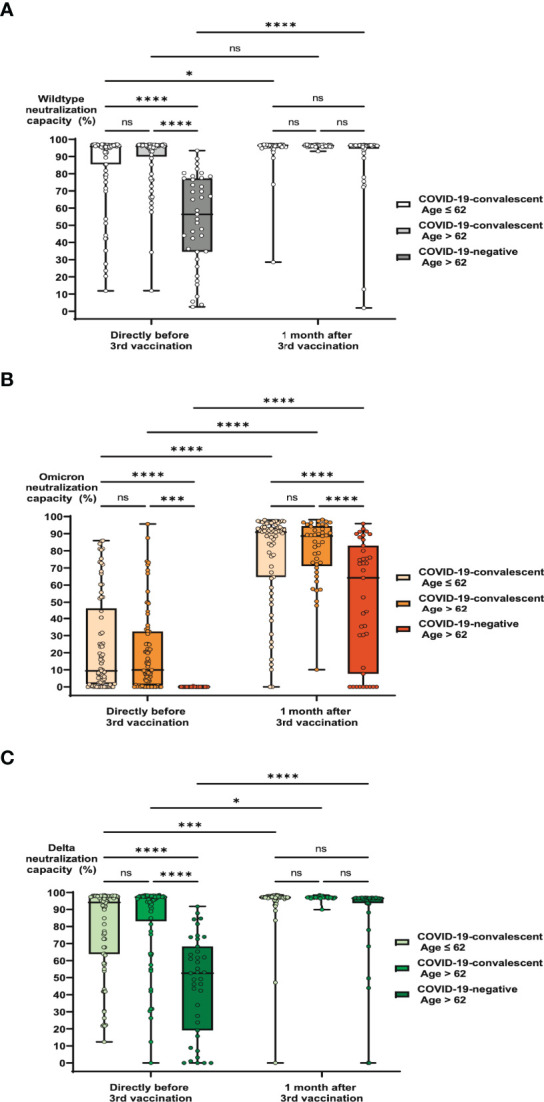
Impact of age and previous SARS-CoV-2 infection on neutralization capacities against variants of concern before and after booster vaccination. Serum samples from 73 COVID-19-convalescent individuals under the age of 63, 74 COVID-19-convalescent individuals above the age of 62 and 39 individuals above the age of 62, who never had a SARS-CoV-2 infection were collected directly before and one month after booster vaccination with BNT162b2. Samples were then analyzed for specific neutralization capacities against wiltype SARS-CoV-2, B.1.1.529 (Omicron) and B.1.617.2 (Delta). Box blots show neutralization capacities in the three study cohorts against **(A)** wild type SARS-CoV-2, **(B)** B.1.1.529 and **(C)** B.1.617.2, before and after vaccination as indicated. Box central horizontal lines indicate medians, box borders represent IQR, whiskers indicate minima and maxima. Significance levels were *p < 0.05, ***p < 0.0005 and ****p < 0.00005. IQR: interquartile ranges, ns: not significant.

Importantly, comparable observations were made with serological neutralization capacities against SARS-CoV-2 including the Omicron and Delta variants. First considering the original wild type SARS-CoV-2 (Wuhan) neutralization capacities, we found that after booster vaccination there were no significant differences between the three cohorts. Again, the elderly COVID-19-naïve cohort appeared to benefit strongest from the booster, with 33% of individuals with strong neutralization capacity (as defined by neutralization > 70%) right before and 95% of individuals after vaccination (**Figure 4A**). Respective percentages in the elderly COVID-19-convalescent cohort raised from 85% before to 98% after third vaccination, those in the younger COVID-19-convalescent cohort from 86% to 100%. After vaccination, no significant differences remained between the cohorts. Although the trends were similar for neutralization capacities against the Omicron variant, the emerging picture was slightly different. While in the elderly COVID-19-naïve cohort not a single individual exhibited any detectable Omicron neutralization capacity in their serum before third vaccination, this percentage raised to 72% after vaccination, of which 46% exhibited strong neutralization capacity (**Figure 4B**). In the COVID-19-convalescent cohorts, which did not show significant age-dependent differences, the percentage of individuals with strong Omicron neutralization capacity ranged between 7 and 14% before third vaccination and reached 98 to 100% after vaccination (**Figure 4B**, **Table 1**). With regard to the Delta variant, results were very similar to wild type SARS-CoV-2. In the elderly COVID-19-naïve cohort, 23% of individuals exhibited strong Delta neutralization capacity right before and 87% of individuals after vaccination (**Figure 4C**). Respective percentages in the elderly COVID-19-convalescent cohort raised from 70% before to 97% after third vaccination, those in the younger COVID-19-convalescent cohort from 80% to 100%.

### Booster Vaccination With BNT162b2 Significantly Enhances SARS-CoV-2-Specific T Cell Responses Irrespective of Age

**Figure 5:**
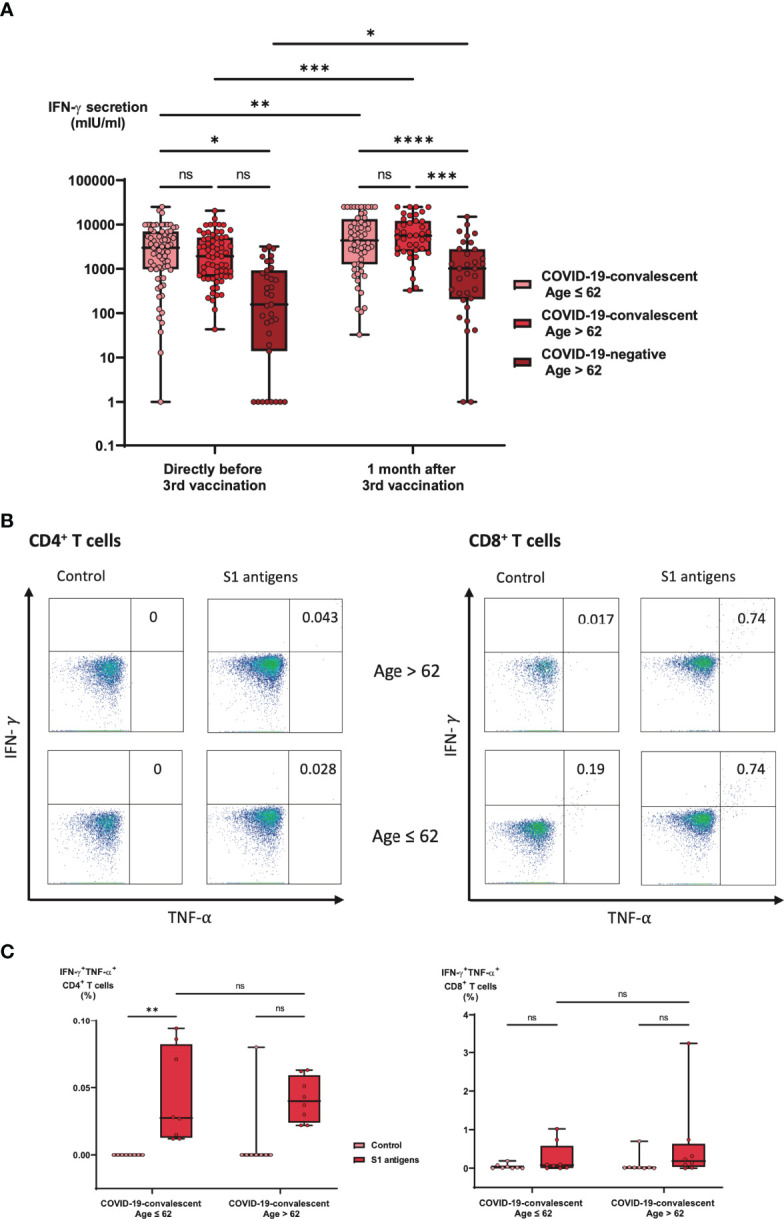
Impact of age and previous SARS-CoV-2 infection on SARS-CoV-2-specific T cell response after booster vaccination. **(A)** Heparin blood samples from 73 COVID-19-convalescent individuals under the age of 63, 74 COVID-19-convalescent individuals above the age of 62 and 39 individuals above the age of 62, who never had a SARS-CoV-2 infection were collected directly before and one month after booster vaccination with BNT162b2. Heparin samples were incubated overnight with a SARS-CoV-2-spike peptide mixture as described in the Methods Section. Then, supernatants were harvested and IFN-g concentrations measured by ELISA. Box blots show IFN-g concentrations in the three study cohorts before and after vaccination as indicated. Box central horizontal lines indicate medians, box borders represent IQR, whiskers indicate minima and maxima. **(B, C)** Heparinized whole blood from COVID-19-convalescent individuals one month after booster vaccination with BNT162b2 was stimulated for 18 hours in both control tubes and Quan-T-cell tubes coated containing a SARS-CoV-2-spike peptide mix as described in the Methods section. After incubation, cells were harvested, stained and analyzed using multi-parameter FACS analysis. Panel **(B)** shows representative percentages of IFN-g^+^ TNF-a^+^ cells in the CD4^+^ T helper (left panel side) and the CD8^+^ cytotoxic T cell (right panel side) populations, both in a convalescent individual > 62 years (upper plots) and a convalescent individual ≤ 62 years of age (lower plots). Box blots in panel **(C)** show average IFN-g^+^ TNF-a^+^ percentages in 8 different individuals > 62 years and in 8 different individuals ≤ 62 years of age. Box central horizontal lines indicate medians, box borders represent IQR, whiskers indicate minima and maxima. Significance levels were *p < 0.05, **p < 0.005, ***p < 0.0005 and ****p < 0.00005. IFN-g, interferon gamma; IQR, interquartile ranges; ns, not significant; S1, spike protein 1; TNF-a, Tumor-necrosis-factor alpha.

In the present study, we also analyzed certain aspects of the cellular immune response in the three different cohorts described above. This included for one part the IFN-γ secretory response of pan T cells specific for an array of wild type SARS-CoV-2 spike peptides. No significant correlations were found between IFN-γ secretion and neutralization capacities, neither against wild type SARS-CoV-2 nor against the Omicron or Delta variants. Nevertheless, overall IFN-γ secretion significantly increased after booster vaccination with BNT162b2 (**Supplementary Figure 3A**) and showed a significant correlation with anti-spike IgG titers (**Supplementary Figure 3B**). Comparison of the three different cohorts demonstrated that no one in the COVID-19-naïve cohort was able to mount a strong IFN-γ response above 4x10^3^ mIU/ml before their third vaccination, while after vaccination a low, but significant 18% of individuals reached such levels (**Figure 5A**). In contrast, in the COVID-19-convalescent cohorts the percentages of individuals with strong IFN-γ response (> 4x10^3^ mIU/ml) before third vaccination ranged between 31 and 42%, and reached between 50 and 62% after third vaccination (**Figure 5A**).

Importantly, for both convalescent groups, the IFN-γ ELISA results were additionally confirmed by FACS-based analysis, which demonstrated that one month after third vaccination with BNT162b2, T cell activation based on the expression of IFN-γ and TNF-α was detectable in both CD4^+^ T helper cells as well as in CD8^+^ cytotoxic T cells (**Figures 5B, C**). Moreover, we found a significant correlation between the percentage of CD4^+^IFN-γ^+^TNF-α^+^ T cells and IFN-γ secretion (r = 0.627, * p > 0.01), whereas the percentage of CD8^+^IFN-γ^+^TNF-α^+^ T cells did not significantly correlate with IFN-γ secretion (r = 0.013, p = 0.96). In line with serological responses, no significant differences were observed between younger and older COVID-19-convalescent individuals (**Figures 5A, C**). Similar results were found when gating on CD45RO^+^ T cells, suggesting that the booster effect of the third vaccination was at least in part based on the activation of a pre-formed memory T cell pool (**Supplementary Figures 4A–C**). This notion was supported by weak but significant correlations between T cell IFN-γ responses before third vaccination and anti-spike IgG titers (**Supplementary Figures 3B**) as well as Omicron neutralization titers (**Supplementary Figure 3C**) one month after third vaccination.

## Discussion

In July 2021, we completed data acquisition for the first part of the AnCORIm study (6). At this time, almost 95% of individuals over the age of 60 years had received at least one vaccination against SARS-CoV-2 in Germany, and more than 75% of this population was fully vaccinated (6th COVIMO report, Robert-Koch-Institute, Germany, August 10th 2021). However, particularly the spreading of the Delta variant fueled concerns that a rapid waning of the anti-SARS-CoV-2 immune response would soon leave vulnerable populations worldwide rather unprotected against this and other newly arising virus variants (24–26). This concern was particularly justified for unvaccinated individuals and COVID-19-naïve individuals vaccinated with a homologous mRNA vaccine approach. In contrast to these two groups, convalescent individuals and individuals receiving heterologous vaccination regimens (e.g. vector + mRNA vaccines) mounted significantly stronger and longer-lasting immune responses against the ancestral SARS-CoV-2 strain as well as the VOCs Alpha, Beta and Gamma (6, 13, 27).

With regard to the waning of the immune response after second vaccination the follow-up study presented here principally confirms the findings from other studies in COVID-19-naïve individuals (24, 25, 26). Our data show a rapid decrease of serological anti-spike antibody responses in COVID-19-convalescent individuals by more than 70% within 5 months after second vaccination with BNT162b2. As anticipated, antibody titers were fully restored one month after booster vaccination in this population, a finding which also translated into a recovery of serological neutralization capacities against the ancestral strain as well as the VOCs Delta and Omicron. Interestingly, already one month after second vaccination with BNT162b2 and four months before the Omicron variant had first been detected in South Africa (28), a significant 82% of convalescent individuals showed detectable and 56% strong neutralization capacity (as defined by neutralization > 70%) against Omicron. Paralleling anti-spike antibody titers, percentages for detectable and strong Omicron neutralization capacity waned over the following 5 months, reaching a nadir at 29% and 10% for detectable and strong neutralization capacity. Then, after booster vaccination, these percentages were again strongly induced and even exceeded those one month after second vaccination, culminating in 92% of individuals exhibiting detectable and 75% strong Omicron neutralization capacity. Similar results were obtained for the Delta variant, although overall neutralization capacities against Delta ranged much closer to those against the ancestral virus with 99% of individuals exhibiting detectable and 98% strong Delta neutralization capacity one month after third vaccination with BNT162b2.

In line with the data from the first part of the AnCORIm study, we did not detect significant differences in serological booster responses in younger versus older convalescent individuals, confirming that age does not appear to be a relevant factor for vaccination responses after successful convalescence from COVID-19 (6). It is noteworthy however that in vaccinated COVID-19-naïve individuals anti-spike antibody titers as well as neutralization capacities ranged significantly lower than in the convalescent cohorts, both after second and after third vaccination with BNT162b2. After booster vaccination this resulted in 95% of individuals exhibiting strong neutralization capacity against the ancestral SARS-CoV-2 strain, 87% against the Delta and only 46% against the Omicron strain.

Particularly our findings regarding the dynamics of the Omicron neutralization capacity impose the interesting hypothesis that Omicron-like mutations may have already broadly occurred on an individual level long before November 2021 (28), since the majority of LTCF outbrakes concerning the participants of our study had happened between March 2020 and February 2021. Even a mildly immunocompromised environment as it may be present in elderly individuals may allow longer and more severe infectious disease courses including COVID-19 (2–4). As described for a patient co-infected with HIV and SARS-CoV-2 (1), this may potentially allow for the temporary emergence and accumulation of additional viral mutations within the infected individual. The participants included in our study had no history of diseases or medications affecting systemic immunity, which may also have a negative impact on vaccination responses (29–34). However, it may be hypothesized that individuals with only mildly compromised immune status can still develop a delayed memory immune response against individually occurring viral mutations, thereby containing and eradicating the corresponding individual viral variants, before further spreading of the virus to persons outside the LTCFs can occur.

A counter-argument against this hypothesis could be that not only unvaccinated convalescent individuals (35), but also BNT162b2-boostered individuals, which are COVID-19-naïve, have been shown to establish some cross-reactive immunity against variants like Omicron (19–21). An explanation for this however may be that all variants described and observed so far including Omicron may have not yet evolved to a point at which they may escape the memory immune responses induced by the currently available vaccines (25). It appears possible therefore that *inactivated whole virus vaccines* could very soon play a so far unforeseen role for COVID-19-naïve individuals who cannot be sufficiently protected against newly arising variants by mRNA vaccines any more. This assumption is also supported by our finding that heterologous vaccination approaches (e.g. ChAdOx1-nCoV-19 and BNT162b2) induce long-lasting and cross-reactive neutralizing immune responses in COVID-19-naïve individuals, which are comparable to those observed in fully vaccinated convalescent individuals (6, 13, 27).

The establishment of an effective and long-lasting immune memory against a virus such as SARS-CoV-2 also requires the presence of an intact cellular immune response during infection or vaccination (14–16, 36). In the present study we demonstrated that this is also the case in COVID-19-convalescent individuals after their second and third vaccination with BNT162b2. The cellular response involved both CD4^+^ and CD8^+^ memory T cells, and as the serological response it appeared to be independent of age. Of note, there was a significant increase in IFN-γ responses between second and third vaccination, suggesting the booster vaccination further fueled the memory effect initiated by the second vaccination. Although a similar increase was observed in the vaccinated COVID-19-naïve control cohort, it was striking that the average cellular response at both time points was more than one log lower than in the vaccinated convalescent cohorts, further supporting the above-discussed differences in quality and quantity of vaccination-triggered immune responses in COVID-19-naïve versus convalescent individuals.

In summary, the second part of our prospective observational cohort study AnCORIm confirmed a general waning of the serological immune response in COVID-19-convalescent individuals 6 months after second vaccination with BNT162b2. As the first part, the second part of our study covered a broad age range between 20 and 98 years at the time of inclusion, with no significant differences regarding immunological response parameters between younger and older convalescent individuals. Importantly, the booster vaccination induced a full recovery of both the cellular immune response and serological response parameters including anti-spike antibody titers with strong neutralization capacities against wild type SARS-COV-2 and the Delta variant. Surprisingly, even serological neutralization capacity against the Omicron variant was detectable one month after second vaccination and four months before it had been first observed in South Africa. As a result, more than 90% of convalescent individuals exhibited detectable and 75% strong Omicron neutralization capacity after booster vaccination with BNT162b2, compared with only 72% and 46% of COVID-19-naïve individuals. Our results support the notion that broad and cross-reactive immune memory against SARS-CoV-2 including the currently known VOCs can be established by booster vaccination with spike-based mRNA vaccines such as BNT162b2, particularly in COVID-19-convalescent individuals of all age groups. Nevertheless, especially in COVID-19-naïve individuals, future variants may escape the memory immune response. In order to finally overcome the SARS-CoV-2 pandemic, such variants may therefore demand additional vaccine approaches such as *inactivated whole virus vaccines*, which include all antigenic components of the virus.

## Data Availability Statement

The raw data supporting the conclusions of this article will be made available by the authors, without undue reservation.

## Ethics Statement

The use of blood from study subjects before and after vaccination was approved by the Ethical Committee at Ulm University. The patients/participants provided their written informed consent to participate in this study.

## Author Contributions

GA and BJ designed the study and performed literature search; GA recruited donors and acquired samples; BJ, MP, JS, JH, CL, CV, and AG performed the analytics; BJ and HS conceptualized and supervised the analytics; MP, JS, JH, CL, CV, and AG collected data, BJ and MP analyzed and interpreted data and prepared figures; BJ, DF, RL, SK, and HS provided key research tools; BJ and GA wrote the manuscript. BJ, MP, DF, and CL verified the underlying data; all authors critically reviewed the manuscript. All authors contributed to the article and approved the submitted version.

## Funding

This work received grant support from the Ministry for Science, Research and Arts of Baden-Württemberg, Germany, and the European Commission (HORIZON2020 Project SUPPORT-E, 101015756) to HS and from the German Red Cross Blood Transfusion Service Baden-Württemberg – Hessen to BJ.

## Conflict of Interest

The authors declare that the research was conducted in the absence of any commercial or financial relationships that could be construed as a potential conflict of interest.

## Publisher’s Note

All claims expressed in this article are solely those of the authors and do not necessarily represent those of their affiliated organizations, or those of the publisher, the editors and the reviewers. Any product that may be evaluated in this article, or claim that may be made by its manufacturer, is not guaranteed or endorsed by the publisher.
