# Supplementary material for: BNT162b2 Booster Vaccination Elicits Cross-Reactive Immunity Against SARS-CoV-2 Variants B.1.1.529 and B.1.617.2 in Convalescents of All Ages
Source: Front Immunol. 2022 Jun 20;13:920210. doi: 10.3389/fimmu.2022.920210 (PMC9250979; doi:10.3389/fimmu.2022.920210)
Supplement: Supplementary file 1 [file DataSheet_1.pdf]

**S1**

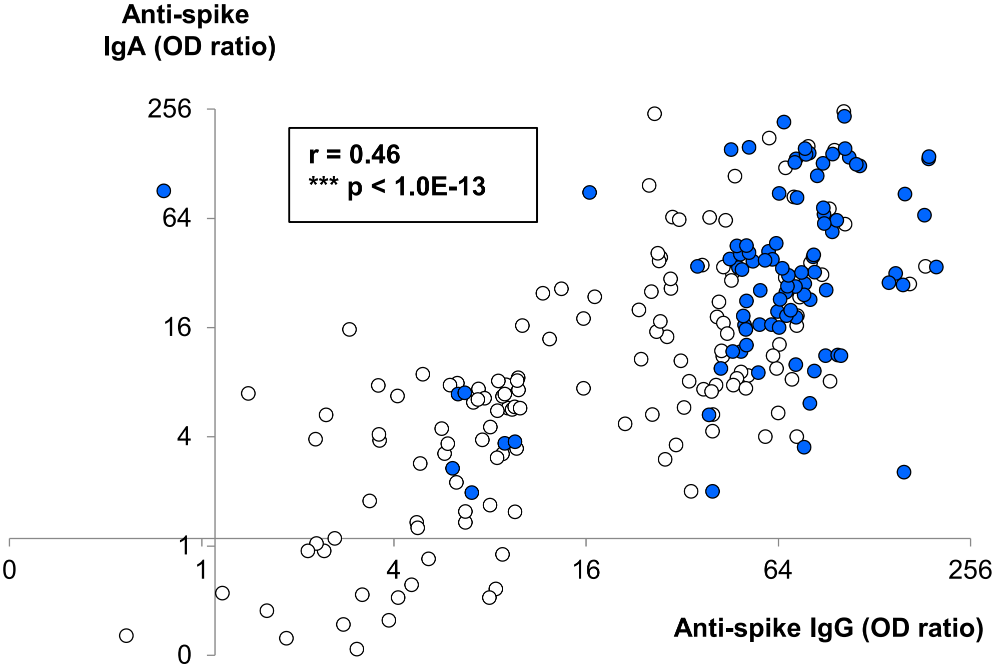


**Supplementary Figure 1. Correlation between anti-spike IgG and IgA titers.**
Serum and heparin samples from up to 147 COVID-19-convalescent subjects were collected directly before and one month after third vaccination with BNT162b2. Then, serum samples were analyzed for anti-spike IgG and IgA titers. Dot plots show correlations between anti-spike IgG and IgA titers with open circles indicating samples before, colored circles samples after third vaccination. P values indicate highly significant Spearman correlation coefficients. Abbreviation: OD: optical density.

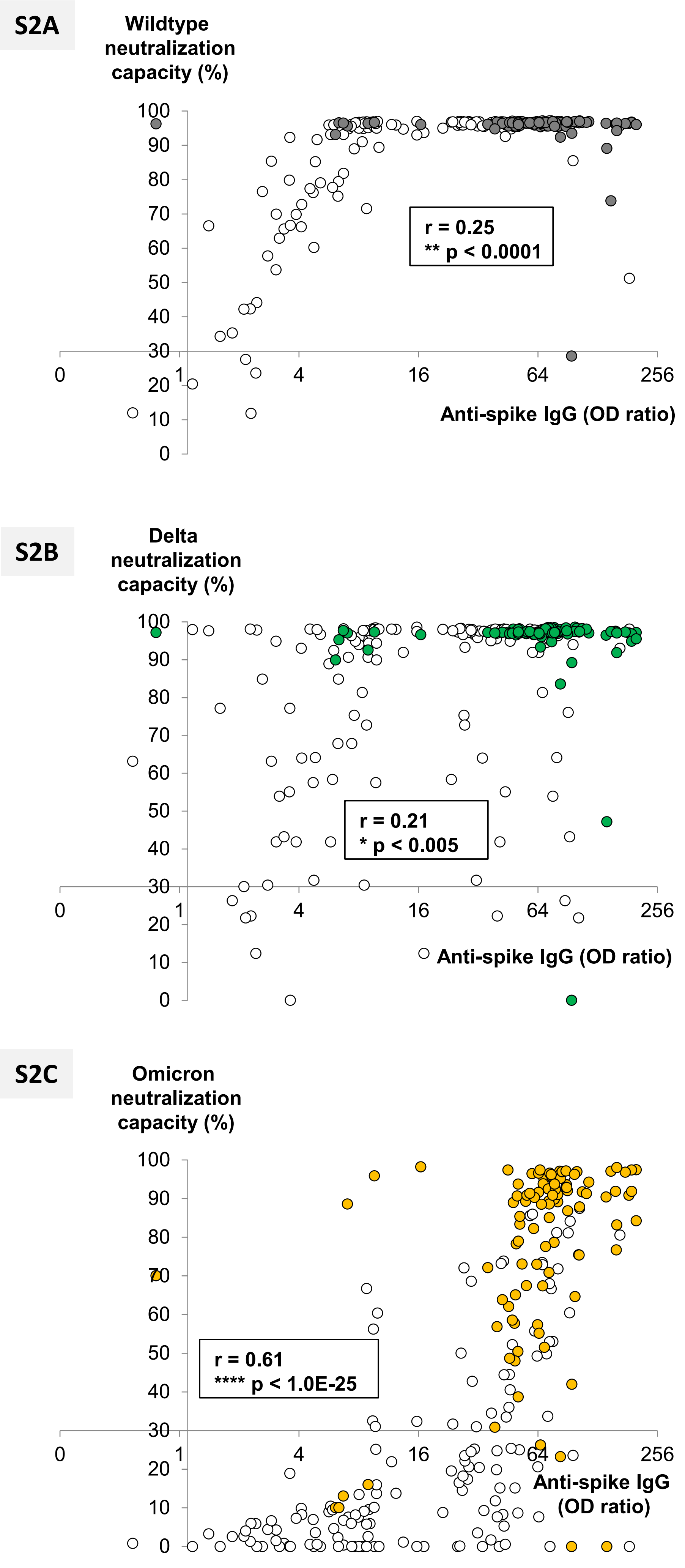


**Supplementary Figure 2. Correlation between anti-spike IgG and neutralization titers.**

Serum samples from up to 147 COVID-19-convalescent subjects were collected directly before and one month after third vaccination with BNT162b2. Samples were then analyzed for anti-spike IgG titers and specific neutralization capacities against wiltype SARS-CoV-2, B.1.617.2 and B.1.1.529. Dot plots show correlations between anti-spike IgG titers and neutralization capacities against (A) wildtype SARS-CoV-2, (B) B.1.617.2 (Delta) and (C) B.1.1.529 (Omicron). Open circles indicate samples before, colored circles samples after third vaccination. P values indicate highly significant Spearman correlation coefficients. Abbreviation: OD: optical density.

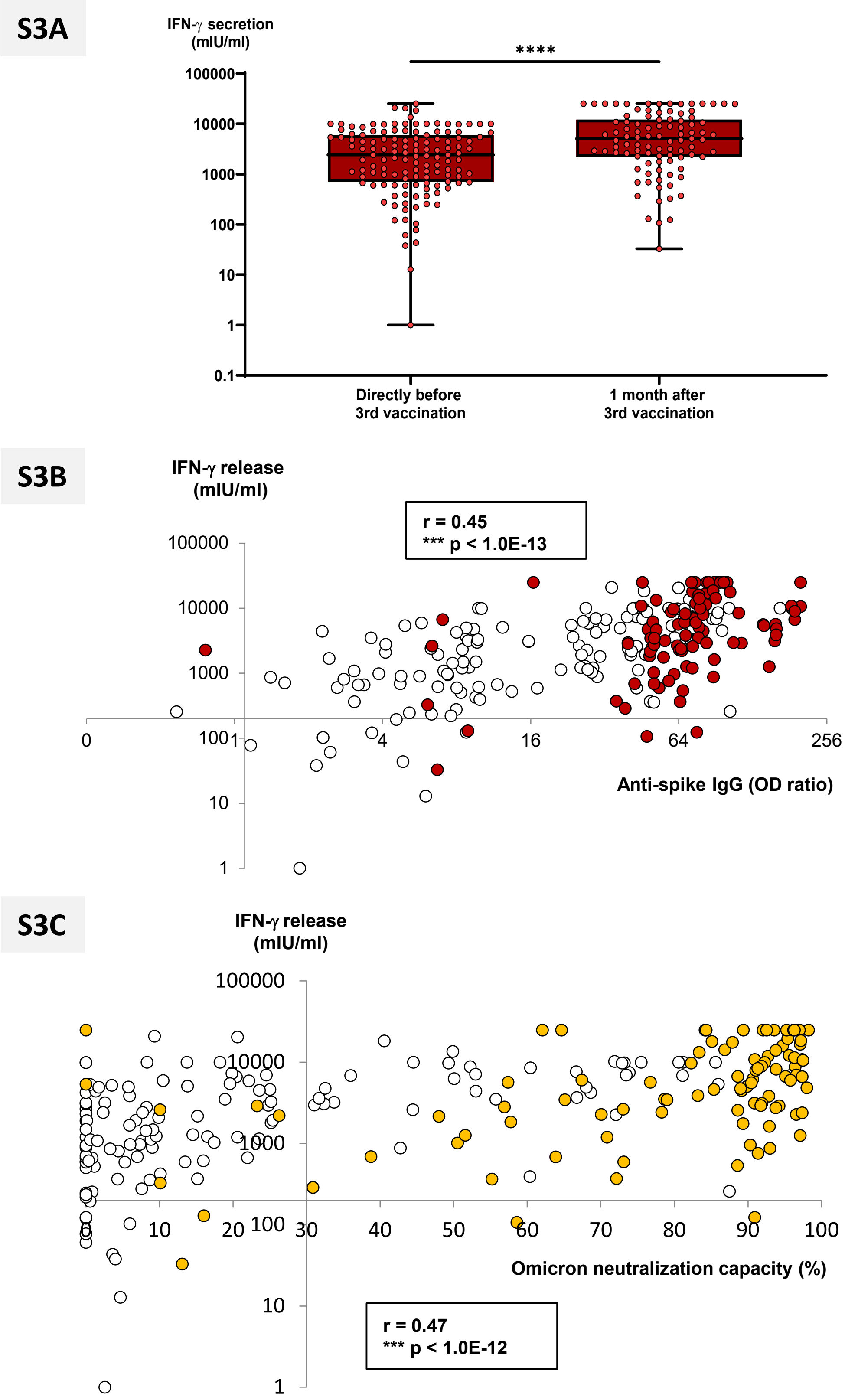


**Supplementary Figure 3. SARS-CoV-2-specific T cell responses before and after booster vaccination and correlation with anti-spike IgG titers.**

Serum and heparin samples from up to 147 COVID-19-convalescent subjects were collected directly before and one month after third vaccination with BNT162b2. Serum samples were analyzed for anti-spike IgG titers and specific neutralization capacity against B.1.1.529. Heparin samples were incubated overnight with a SARS-CoV-2-spike peptide mix and IFN-γ concentrations were analyzed by ELISA. (A) Box blots show IFN-γ concentrations before and after vaccination as indicated. Box central horizontal lines indicate medians, box borders represent IQR, whiskers indicate minima and maxima. Significance level was **** $p < 0.00005$. (B) Dot plots show a significant correlation between anti-spike IgG titers and IFN-γ concentrations. (C) Dot plots show a significant correlation between neutralization capacities against B.1.1.529 (Omicron) and IFN-γ concentrations. Open circles indicate samples before, colored circles samples after third vaccination. P values indicate highly significant Spearman correlation coefficients. Abbreviations: IFN-γ: interferon gamma, OD: optical density, IQR: interquartile ranges.

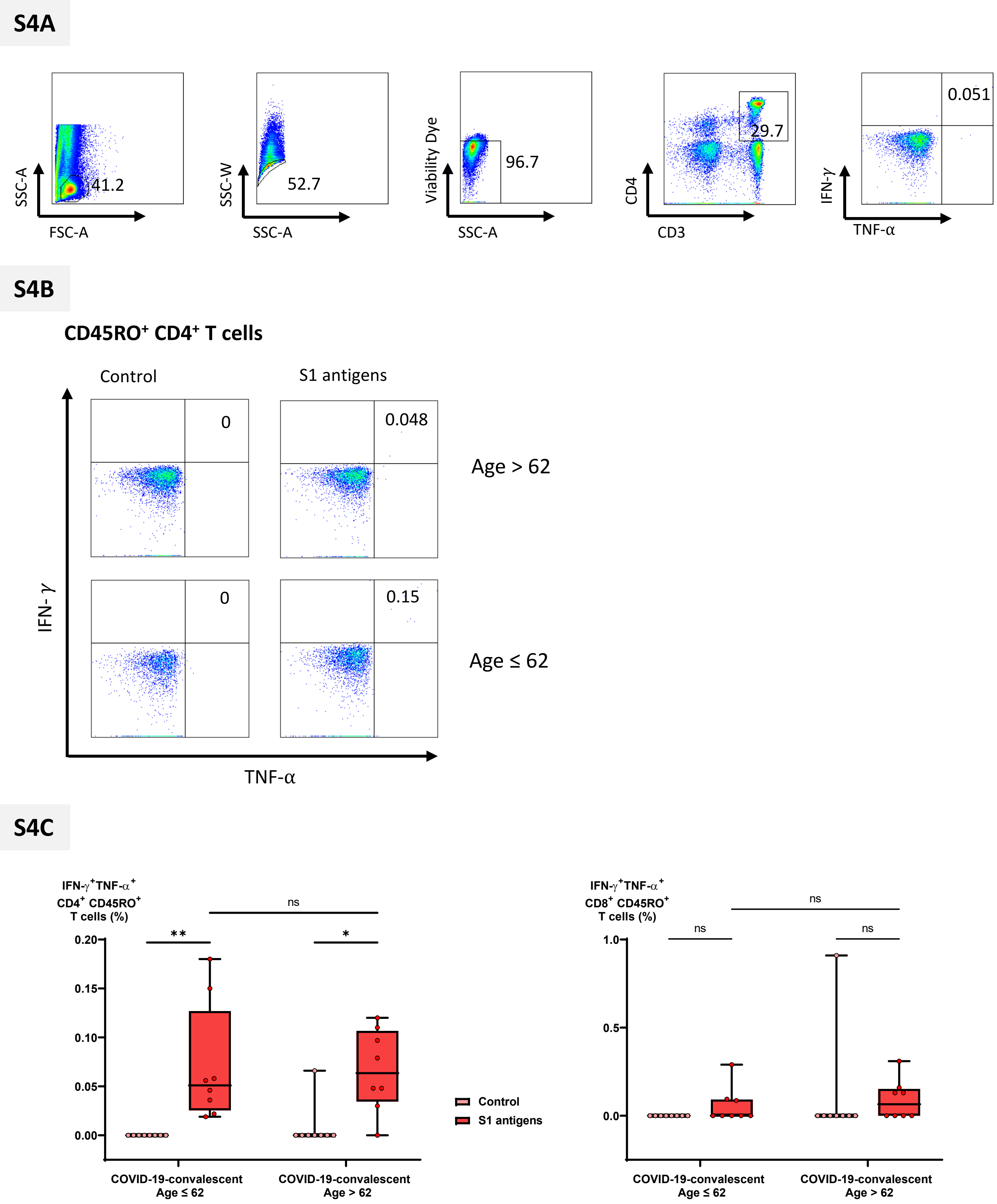


**Supplementary Figure 4. Differential SARS-CoV-2-spike-specific memory T cell responses after vaccination.**
Heparinized whole blood from COVID-19-convalescent individuals one month after booster vaccination with BNT162b2 was stimulated for 18 hours in both control tubes and Quan-T-cell tubes coated with an S1 peptide mixture as described in Materials and Methods. After incubation, cells were harvested, stained and analyzed using multi-parameter FACS analysis. Panel (A) shows the gating strategy, panel (B) shows representative percentages of IFN-$\gamma^+$ TNF-$\alpha^+$ cells in the CD45RO$^+$ CD4$^+$ memory T helper cell population, both in a convalescent individual > 62 years (upper plots) and a convalescent individual ≤ 62 years of age (lower plots). Box blots in panel (C) show average IFN-$\gamma^+$ TNF-$\alpha^+$ percentages in 8 different individuals > 62 years and in 8 different individuals ≤ 62 years of age, both for the CD4$^+$ T helper (left panel side) and the CD8$^+$ cytotoxic T cell (right panel side) populations. Box central horizontal lines indicate medians, box borders represent IQR, whiskers indicate minima and maxima. Significance levels were * $p < 0.05$ and ** $p < 0.005$. Abbreviations: IFN-γ: interferon gamma; ns: not significant; S1: spike protein 1; TNF-α: Tumor-necrosis-factor alpha.
